# Smoking, Antioxidant Supplementation and Dietary Intakes among Older Adults with Age-Related Macular Degeneration over 10 Years

**DOI:** 10.1371/journal.pone.0122548

**Published:** 2015-03-30

**Authors:** Bamini Gopinath, Victoria M. Flood, Annette Kifley, Gerald Liew, Paul Mitchell

**Affiliations:** 1 Centre for Vision Research, Department of Ophthalmology and Westmead Millennium Institute, University of Sydney, Sydney, Australia; 2 Faculty of Health Sciences, University of Sydney and St Vincent’s Hospital, Sydney, Australia; Copenhagen University Hospital Roskilde and the University of Copenhagen, DENMARK

## Abstract

We aimed to compare the micronutrient usage and other lifestyle behaviors over 10 years among those with and without age-related macular degeneration (AMD). 1612 participants aged 49+ years at baseline were re-examined over 10 years, west of Sydney, Australia. AMD was assessed from retinal photographs. Dietary data were collected using a semi-quantitative food frequency questionnaire. Smoking status was self-reported. 56 participants had any AMD at baseline, of these 25% quit smoking at 5 years and were still not smoking at 10-year follow-up. Among participants who had below the recommended intake of vitamins A, C or E supplements at baseline, those who did compared to those who did not develop late AMD over 10 years were more likely to report vitamins A (total), C or E supplement intake above the recommended intake at 10-year follow-up: multivariable-adjusted OR 4.21 (95% CI 1.65-10.73); OR 6.52 (95% CI 2.76-15.41); and OR 5.71 (95% CI 2.42-13.51), respectively. Participants with compared to without AMD did not appreciably increase fish, fruit and vegetable consumption and overall diet quality. Adherence to smoking and dietary recommendations was poor among older adults with AMD. However, uptake of antioxidant supplements increased significantly among those with late AMD.

## Introduction

Age-related macular degeneration (AMD) is a leading cause of irreversible blindness in older adults of developed countries [1,2]. The current treatment modalities are only appropriate and partially effective for a subgroup of patients [3]. Preventive strategies through dietary modulation are attractive strategies, because many studies suggest benefit of certain micro- and macro-nutrients with respect to AMD. The Age-Related Eye Disease Study (AREDS) showed less progression from early to late stage AMD with high-dose zinc/antioxidant supplement compared to placebo [4–9]. A follow-up study, AREDS 2, also showed benefits from dietary consumption of lutein/ zeaxanthin but not with long-chain omega-3 fatty acid supplementation [10,11].

In spite of the potential for considerable public health impact, low adherence to use of AREDS supplements in patients with AMD has been reported [3,12–14]. A US cross-sectional clinical case series of 332 adults with AMD, more than a third of those deemed candidates for AREDS-type supplements were not using them or using an incorrect dose [13]. However, in a more recent US cross-sectional study of 92 AMD patients, adherence to: AREDS supplementation, dietary modification and exercise/weight reduction was 88%, 81% and 76%, respectively. None of the participants adhered to advice on smoking cessation [12].

There is a paucity of cohort studies that have compared the micronutrient usage and other modifiable lifestyle behavior patterns in the longer term among those with and without a diagnosis of AMD. Therefore, the key study questions were: 1) Is there an appreciable decrease in the smoking rates among participants presenting with AMD at baseline? 2) Does antioxidant supplement use differ significantly between older adults with and without AMD over a 10-year period? 3) Is consumption of fish, fruit and vegetables over 10 years significantly different between participants with and without AMD? and 4) Are there appreciable changes in 10-year diet quality following AMD presentation at baseline?

## Methods

### Study population

The Blue Mountains Eye Study (BMES) is a population-based cohort study of common eye diseases and other health outcomes in a suburban Australian population located west of Sydney. Study methods and procedures have been described elsewhere [15]. Baseline examinations of 3654 residents aged >49 years were conducted during 1992–4 (BMES-1; 82.4% participation rate). Surviving baseline participants were invited to attend examinations after 5- (1997–9, BMES-2), 10- (2002–4, BMES-3), and 15 years (2007–9, BMES-4) at which 2334 (75.1% of survivors), 1952 participants (75.6% of survivors) and 1149 (55.4% of survivors) were re-examined, respectively. For the current report we have analyzed data from BMES-1 through to -3. The University of Sydney and the Western Sydney Area Human Ethics Committees approved the study, and written, informed consent was obtained from all participants at each examination.

### Assessment of AMD

We took two 30° stereoscopic color retinal photographs of the macula of both eyes, which were graded for presence of early and late AMD using the Wisconsin AMD Grading System [16,17]. Inter- and intra-grader reliability showed good agreement for grading of specific AMD lesions with quadratic weighted kappa values ranging from 0.64 to 0.93 and 0.54–0.94 respectively [18]. The detailed methodology of AMD ascertainment in this population has been previously reported [16,17]. Early AMD was defined as the absence of late AMD and presence of either: 1) large (>125-μm diameter) indistinct soft or reticular drusen or 2) both large distinct soft drusen and retinal pigmentary abnormalities (hyperpigmentation or hypopigmentation) in either eye [17]. Similarly, late AMD was defined as the presence of neovascular AMD or geographic atrophy in either eye [17]. Any AMD was defined as having early or late AMD. A retinal specialist (P.M.) adjudicated all uncertain retinal pathology and confirmed all late AMD cases. Patients and their physicians were informed by mail if they were diagnosed with AMD through these photographs

### Assessment of diet

Dietary data were collected using a 145-item self-administered food frequency questionnaire (FFQ) at BMES-1, -2 and -3. The FFQ is modified for Australian diet and vernacular from an early Willett FFQ [19] and including reference portion sizes. The FFQ was validated by comparing nutrients from the FFQ to 3 x 4-day weighed food records collected over one year (n = 79), in order to assess seasonal variation. Most nutrient correlations were between 0.50 and 0.60 for energy-adjusted intakes, similar to other validated FFQ studies [20,21]. A dietician coded data from the FFQ into a customized database that incorporated the Australian Tables of Food Composition 1990 (NUTTAB 90) and follow-up data used NUTTAB95 [22,23]. Foods listed in the FFQ were categorized into major food categories and subcategories similar to those used for the 1995 Australian National Nutrition Survey [24]. We also extracted separate data on the frequency of consuming fish and oily fish (salmon, tuna and sardines). The cut-points used for analyses of fruit, vegetable and fish consumption were based on the overall recommended daily intake (RDI) for each food group [25]. These cut-points were: ≥375 g/ day for vegetables (five 75 gram serves/ day); ≥300 g/ day for fruit (two 150 gram serves/ day); and ≥28.5 g/ day for fish (two 100 gram serves/ day) [25].

The vitamin and mineral supplement section of the FFQ included questions about brand name, frequency, strength, and duration of use for multivitamin preparations and individual supplements (e.g. vitamins A, C, E, beta-carotene and zinc) [26]. Total Vitamin A in microgram retinol equivalents was estimated from retinol μg + (beta-carotene μg/6), based on Nutrient Reference Values for Australia and New Zealand [27]. The cutpoints for intake of total vitamin A, vitamin C and zinc supplements were based on the overall RDI for each nutrient [27]. The cutpoints for retinal or beta-carotene supplements are based on the RDI for total vitamin A because there is no separate guideline for subcomponents of total Vitamin A [27]. The cutpoint for vitamin E supplements is based on adequate intake (AI) because an RDI is not clearly established. These cutpoints are vitamin A—700 μg/ day in women and 900 μg /day in men; vitamin C—45 mg/ day in men or women; vitamin E—10 mg/ day in men and 7 mg/ day in women; beta-carotene—5400 μg/ day in men and 4200 μg/ day in women; and zinc—14 mg/ day in men and 8 mg/ day in women [27].

A modified version of the Australian diet quality index [28], based on the Dietary Guidelines for Australian Adults (DGAA) [25] and the Australian Guide to Healthy Eating [29], was used to establish the total dietary score, assessing adherence to the Australian dietary guidelines. The methodology used to develop total dietary scores has been previously reported [30]. Briefly, total dietary scores were allocated for intake of selected food groups and nutrients for each participant as described in the DGAA. The total dietary score is divided into ten components, and each component has a possible score ranging from 0 to 2. A maximum score of 2 was given to subjects who met the recommendations with prorated scores for lower intakes. These were then summated providing a final score ranging between 0 and 20 with higher scores indicating closer adherence to the dietary guidelines.

### Assessment of other lifestyle behaviors

At face-to-face interviews with trained interviewers, a comprehensive medical history that included information about demographic factors, socio-economic characteristics and lifestyle factors such as smoking, was obtained from all participants. Smoking status was determined from history as never smoked, past smoker, and current smoker. Participants were also asked about their education level and whether they received a pension and the type. Body mass index (BMI) was calculated as weight divided by height squared (kg/m^2^).

### Statistical analysis

SAS statistical software (SAS Institute, Cary NC) version 9.2 was used for analyses. Baseline characteristics of study participants who were followed over 10 years were compared using t-tests and χ^2^-tests. The association with smoking cessation involved analyses of baseline AMD status. While all other analyses (i.e. associations with antioxidant supplement usage and dietary parameters) involved cumulative AMD cases, which was defined as the presence of AMD at either BMES 1, 2 or 3. The frequency of smoking cessation at 5 or 10 years was cross-tabulated by AMD status among participants who reported current smoking at baseline. Frequency tables and χ^2^-tests of independence were used to assess whether participants who were below the recommend intake for each antioxidant supplement at baseline were more likely to be at or above recommended intakes after 10 years if they had evidence of AMD at baseline, 5 or 10 years (i.e. cumulative cases), compared to those who did not have evidence of AMD at any time point. Separate analyses were conducted for early AMD and late AMD, and for each of the antioxidant supplements: vitamins A (retinol and beta-carotene), C and E, and zinc. Similarly, the association between early and late AMD and the probability of being below the RDI for vegetables, fruits or fish intake at baseline to being at or above RDI at 10 years, and from low to high total diet scores were examined. Logistic regression was used to confirm any significant associations after adjusting for age, gender, past/current smoking, body mass index, receipt of a pension, and post-school qualifications, with findings presented as adjusted odds ratios (OR) and 95% confidence intervals (CI).

## Results

Of the 3654 BMES participants at baseline, 1612 participated in BMES 1, 2 and 3, and had complete information on AMD status at baseline. Hence, 1612 participants were included in the analyses. Participants with any AMD compared to those without AMD were older and more likely to receive the age pension (Table 1). Of the 56 AMD cases at baseline, 47 were early AMD and 9 were late AMD cases. There were no significant differences in 10-year smoking cessation rates between participants with (n = 56) and without AMD (n = 1556) who reported that they currently smoked at baseline (S1 Fig). Specifically, 25% and 20% of those with and without any AMD, respectively, quit smoking at 5 years and continued to not smoke at 10 year follow-up.

**Table 1:** Study characteristics of participants stratified by AMD status at baseline who were followed up at the 10-year follow up in 2007–9 Blue Mountains Eye Study.

Characteristics	No AMD (n = 1556)	Any AMD (n = 56)	P-value
Age	62.3 (7.5)	70.8 (7.9)	<0.0001
Male sex	638 (41.0)	21 (37.5)	0.60
High school qualifications	936 (62.8)	27 (51.9)	0.11
Receipt of pension	698 (46.2)	36 (66.7)	0.003
Current smoker	176 (11.6)	8 (14.6)	0.51
Body mass index	26.4 (4.3)	25.6 (3.8)	0.18
Healthy eating index score	11.2 (2.3)	11.3 (2.2)	0.78
Fruits, *g/day*	355.5 (272.2)	415.6 (309.1)	0.14
Vegetables, *g/day*	445.4 (186.9)	443.4 (153.7)	0.94
Fish, *g/day*	26.9 (25.1)	27.3 (25.3)	0.93
Antioxidant supplement use			
Total Vitamin A	177 (13.1)	4 (8.5)	0.36
Vitamin C	421 (31.1)	11 (23.4)	0.26
Vitamin E	268 (19.8)	11 (23.4)	0.54
β-carotene	79 (5.8)	3 (6.4)	0.87
Zinc	196 (14.5)	5 (10.6)	0.46

Data are presented as n (%) or mean (SD)

There were 198 and 57 cumulative cases of early and late AMD, respectively, across the 3 examinations or over 10 years (i.e. from BMES-1 to BMES-3). Among participants who developed early AMD at either the baseline, 5 or 10-year follow-up (i.e. cumulative cases) and were below recommended intakes for vitamins E, A (total) and C, and zinc supplement use at baseline: 14%, 11%, 21%, and 10% were at or above recommended intakes 10 years later, respectively. Among cumulative late AMD cases: 37%, 23%, 46%, and 25% were at or above recommended intakes for vitamins E, A and C, and/or zinc supplements at 10 years, respectively.

Among participants who were below RDI for zinc at baseline, those with than without cumulative early AMD were ~2-fold more likely to report zinc supplement use at or above RDI after 10 years (Table 2). Among participants who were below the RDI for vitamins A (total), C or E at baseline, those with compared to without cumulative late AMD were 4-, 6- and 5-fold more likely to report vitamins A, C or E supplement use at or above RDI, respectively, after 10 years. Cumulative cases of late AMD were also positively associated with β-carotene and zinc supplement uptake over 10 years. Supplementary analyses involved examining changes in intake of an ‘AREDS-style’ formula, which we defined as 25 mg zinc plus any level of either vitamin C or vitamin E. We found that those with compared to without cumulative late or early AMD were more likely to take up this ‘AREDS-style’ formula over the 10 years, multivariable-adjusted OR 14.7 (95% CI 4.94–43.9) and OR 3.96 (95% CI 1.68–9.30), respectively.

**Table 2:** Association between cumulative AMD and incident antioxidant supplement usage over 10 years ^a^ in the Blue Mountains Eye Study, presented as adjusted odds ratio (OR) and 95% confidence intervals (CI).

		Antioxidant supplement uptake, OR (95% CI)^b^				
Cumulative AMD	Vitamin E	Retinol	β-carotene	Total vitamin A^c^	Vitamin C	Zinc
Early AMD^d^	1.13 (0.61–2.12)	2.01 (0.97–4.17)	0.88 (0.29–2.62)	1.71 (0.88–3.33)	1.54 (0.87–2.72)	2.55 (1.27–5.10)
Late AMD^d^	5.71 (2.42–13.51)	2.41 (0.77–7.54)	4.26 (1.13–16.08)	4.21 (1.65–10.73)	6.52 (2.76–15.41)	8.52 (3.36–21.57)

^a^ Analyses included participants who were below the recommended daily intake for vitamins A, C, or E or β-carotene, and/or zinc at baseline.

^b^ Adjusted for age, sex, smoking, education, BMI, and receipt of pension

^c^ Total Vitamin A in μg retinol equivalents = retinol micrograms + (beta-carotene μg / 6)

^d^ Cumulative cases of early or late AMD over the 10-year follow-up

Among those who were below the RDI for fruit and vegetables at baseline, those who did not compared to those who developed late AMD over 10 years were more likely to eat fruits at BMES-3 (Table 3). There were no significant differences in the number of participants who developed early or late AMD compared to those with no AMD at either baseline, 5- or 10-year follow-up who went from a lower (<10.5) to a higher total diet score (≥10.5) after 10 years, p = 0.6 and p = 0.9, respectively.

**Table 3:** Association between cumulative cases of AMD and increase in consumption of fruits, vegetables (in g/day), and fish over 10 years in the Blue Mountains Eye Study.

	At or above recommended intake at BMES-3 following lower intake at BMES-1, n (%)		
Cumulative AMD	Without AMD	With AMD	P-value
Early AMD			
Vegetables ^a^	161 (44.4)	23 (45.1)	0.9
Fruits ^b^	159 (32.9)	15 (25.9)	0.2
Fish ^c^	247 (36.7)	42 (42.9)	0.2
Late AMD			
Vegetables ^a^	200 (44.6)	5 (41.7)	0.8
Fruits ^b^	189 (32.5)	1 (4.7)	0.03
Fish ^c^	306 (37.3)	8 (33.3)	0.7

^a^ At or above recommended vegetable intake at BMES-3 defined as ≥375 g/day (five 75 gram serves per day) and lower intake at BMES-1 as <375 g/day

^b^ At or above recommended fruit intake at BMES-3 defined as ≥300 g (two 150 gram serves per day) and low intake at BMES-1 as <300 g

^c^ At or above recommended fish consumption at BMES-3 defined as ≥28.5 g/day (two 100 gram serves per day) and low consumption at BMES-1 as <28.5 g/day

## Discussion

This novel cohort study provides a better understanding of the change in modifiable lifestyle behaviors among older adults with AMD. Around half of older adults with any AMD at baseline had not quit smoking over the 10-year period. Participants who did develop late AMD compared to those who did not develop late AMD at either of the examinations, were more likely to take up antioxidant supplements at the 10-year examination. A lower proportion of older adults with versus without late AMD reported high fruit consumption at 10-year follow-up. Also, participants with AMD did not appreciably increase their diet quality over the 10 years.

Smoking is an established AMD risk factor, and its cessation is an important recommendation with the potential to alter AMD progression [12,31]. However, in our study only one in four current smokers with any AMD at baseline quit smoking at 5years and were still not smoking at 10 years, with the remaining 50% still smoking after 10 years. This concurs with the finding that despite Australia’s relative success in educating smokers about the association between smoking and ocular diseases, ~30% of adults smokers (particularly older adults) were either unaware or had difficulty understanding the effects of smoking on ocular health [32]. These findings highlights these as important issues for eye healthcare providers to consider, who are in a unique position to help educate patients about the health effects, and to support national prevention and cessation efforts [32]. Appropriate strategies could include encouraging ophthalmologists to be more active in asking a subgroup of their high-risk patients if they smoke; give brief advice to quit and refer them to a smoking cessation specialist.

Despite the potential for public health impact, low adherence to use of AREDS-type supplementation in patients with AMD has been previously reported [3,12–14]. A US [14] and Australian study [3] showed that only 42.5% and 38% of AMD patients, respectively, were following recommendations for AREDS-like supplementation. The rates from these studies are similar to the low rates in the present study, with ~19%-46% of older adults who developed late AMD over 10 years taking up antioxidant supplementation. Hence, this study highlights the presence of a substantial gap between recommendations made by eye healthcare specialists and the recommendations actually practiced by AMD patients [12]. Our study does not allow us to determine the reasons for poor adherence to antioxidant supplementation recommendations, but it is likely to be multifactorial in origin [12]. Previous studies reported that some of the common reasons that AMD patients did not adhere to antioxidant supplement recommendations are: that they were most often unaware of recommendations to do so; and personal questions regarding benefit and supplement cost [3,14]. Although, it is unlikely to be a cost-related issue as we had adjusted for socio-demographic indicators such as receipt of pension payment.

Cumulative cases of early AMD was only associated with the significant uptake of zinc supplements at 10 year follow-up, while cumulative late AMD cases were associated with a greater likelihood of taking up all of the antioxidant supplements assessed in this study. We speculate that persons with advanced AMD could have been symptomatic or more concerned by their macula health and the prospects of going blind than a person with milder disease or in the earlier stages of AMD, and thus, would have been more inclined to adhere to recommendations for antioxidant supplementation during the 10 years. The lower uptake of antioxidant supplements in those who developed early AMD could be a cause for concern given that it is this sub-group which is at greatest risk of progression to advanced AMD and thus, irreversible vision loss [33]. Moreover, there are few effective therapeutic strategies available that target early AMD signs [34]. Hence, this finding highlights the need for educational strategies that target those with early AMD lesions in order to improve compliance to recommendations for antioxidant supplement use.

Recent data from BMES and Rotterdam Study demonstrate that carriers of certain complement factor-H and ARMS2 gene polymorphisms increase late AMD risk 2-4-fold, but may substantially reduce their risk to close to non-carrier levels by regular consumption of fish [35], omega-3 fatty acids, zinc/antioxidants [36]. Thus, AMD gene carriers may be able to 'eat away their genetic risk'. However, we show that older adults with AMD were not increasing their consumption of vegetables over the 10 years, even though vegetables are a rich source of lutein/ zeaxanthein. Moreover, we show that older adults who developed AMD were not increasing their diet quality. The lack of adherence to these lifestyle behavioral recommendations, could be due to patient failure to understand the importance of AMD-related recommendations, inadequate explanation and reinforcement from ophthalmologists, and lack of nutritionists support and referral [12]. Therefore, this study underscores the importance of proper patient education of the lifestyle recommendations and regular follow-up by the appropriate clinicians to ensure that these recommendations are being implemented routinely and correctly [12].

Strengths of this study include its prospective design, and robust data on major confounders and dietary parameters. Further, this study uses high quality stereoscopic retinal photography with validated grading to assess macular conditions, and a detailed side-by-side comparison of the baseline and follow-up photographs to ensure negligible misclassification of incident AMD [37,38]. However, this study is limited by the relatively few incident AMD cases over the 10-year follow-up. Therefore, we may not have sufficient power to detect modest associations between the development of AMD and change in lifestyle behaviors. Additionally, dietary assessment by FFQ in which respondents have to estimate typical intake frequencies of food items and their portion sizes can potentially introduce measurement error and recall bias. A further limitation is the use of a survivor cohort, for instance persons with AMD who may have quit smoking or improved their diet quality could have been lost to follow-up or have died (as they are on average ~8 years older than those without AMD at baseline), before any modifications to lifestyle behaviors could be documented.

Despite the shortfalls, this study provides unique data on change (or lack thereof) in modifiable lifestyle behaviors among older adults with and without AMD. These findings suggest that current smoking cessation strategies are not efficaciously targeting older, current smokers with AMD. Similarly, older adult with compared to without AMD are not more likely to adhere to dietary recommendations. Although, rate of antioxidant supplement use was low in our cohort, it was encouraging to observe the appreciable increase in uptake of antioxidants supplements among those with compared to without late AMD. In summary, these study findings highlights the importance of intensifying patient educational efforts by eye healthcare providers regarding the ophthalmologic and public health benefits of smoking cessation, antioxidant supplementation and optimal diet quality.

## Supporting Information

S1 Fig Quit rates among current smokers with and without AMD at baseline examination. No significant differences were found in quit rates during the 10-year follow-up between current smokers at baseline who did or did not have any AMD. Unsustained smoking cessation means that they tried to quit but then resumed again sometime over the 10 years (i.e. quit smoking at the 5-year follow-up but resumed again at the 10-year follow-up).
(TIFF)

S1 Dataset Study participant dataset used for analyses in this report (XLS)
