# Supplementary figures and images for: Smoking, Antioxidant Supplementation and Dietary Intakes among Older Adults with Age-Related Macular Degeneration over 10 Years
Source: PLoS One. 2015 Mar 30;10(3):e0122548. doi: 10.1371/journal.pone.0122548 (PMC4378919; doi:10.1371/journal.pone.0122548)

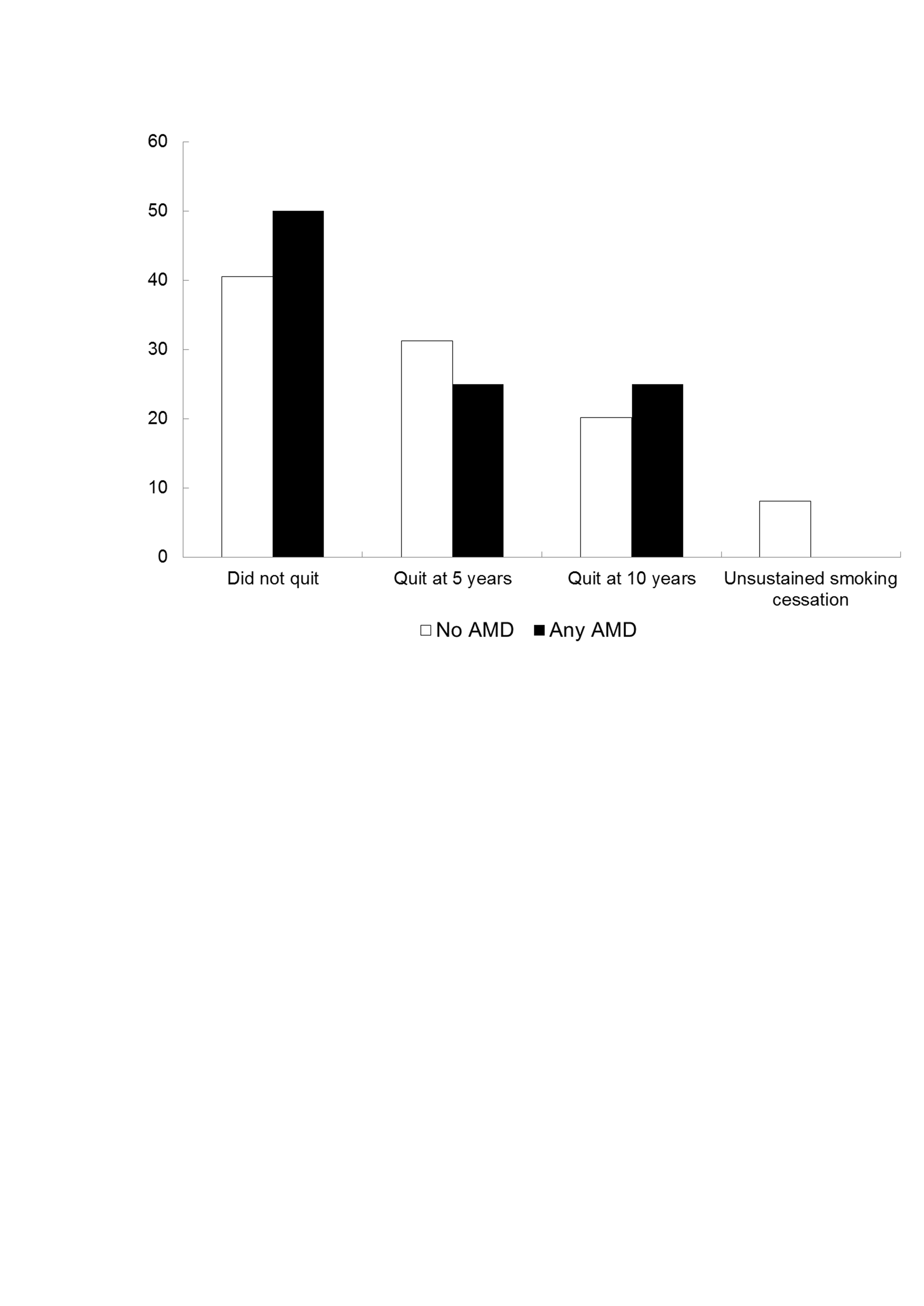

Supplement: S1 Fig — No significant differences were found in quit rates during the 10-year follow-up between current smokers at baseline who did or did not have any AMD. Unsustained smoking cessation means that they tried to quit but then resumed again sometime over the 10 years (i.e. quit smoking at the 5-year follow-up but resumed again at the 10-year follow-up). (TIFF) [file pone.0122548.s001.tiff]
